# Visualization of Microfloral Metabolism for Marine Waste Recycling

**DOI:** 10.3390/metabo6010007

**Published:** 2016-01-27

**Authors:** Tatsuki Ogura, Reona Hoshino, Yasuhiro Date, Jun Kikuchi

**Affiliations:** 1 RIKEN Center for Sustainable Resource Science, 1-7-22 Suehiro-cho, Tsurumi-ku, Yokohama 230-0045, Japan; tatsuki.ogura@riken.jp (T.O.); yasuhiro.date@riken.jp (Y.D.); 2 Graduate School of Medical Life Science, Yokohama City University, 1-7-29 Suehiro-cho, Tsurumi-ku, Yokohama 230-0045, Japan; r-hoshino.mf2@tsurumi.yokohama-cu.ac.jp; 3 Graduate School of Bioagricultural Sciences, Nagoya University, 1 Furo-cho, Chikusa-ku, Nagoya 464-0810, Japan

**Keywords:** marine waste, anaerobic fermentation, soil amendment, NMR

## Abstract

Marine biomass including fishery products are precious protein resources for human foods and are an alternative to livestock animals in order to reduce the virtual water problem. However, a large amount of marine waste can be generated from fishery products and it is not currently recycled. We evaluated the metabolism of digested marine waste using integrated analytical methods, under anaerobic conditions and the fertilization of abandoned agricultural soils. Dynamics of fish waste digestion revealed that samples of meat and bony parts had similar dynamics under anaerobic conditions in spite of large chemical variations in input marine wastes. Abandoned agricultural soils fertilized with fish waste accumulated some amino acids derived from fish waste, and accumulation of l-arginine and l-glutamine were higher in plant seedlings. Therefore, we have proposed an analytical method to visualize metabolic dynamics for recycling of fishery waste processes.

## 1. Introduction

The new millennium is the era to make efficient use of marine biomass by cultivation of the ocean although humans have been harvesting on land with terrestrial animal extinctions in human history up until now [1]. The ocean covers approximately 70% of the earth’s surface and produces many diverse and precious marine biomasses, including healthy food products that are consumed on a daily basis. In particular, fishery products such as fish and shellfish are precious protein resources that have a reduced virtual water load compared with livestock animals; thus, utilization of marine biomasses is considered as one possible solution to address global food shortages [2,3,4]. Japan has utilized marine biomasses as an alternative to livestock animals for many years [5]; however, this is accompanied by the production of tons of waste, most of which is currently not recycled. Therefore, the utilization and recycling of these wastes are important issues to address.

Marine waste includes seashells, exoskeletons of crustaceans, and the bony parts of fish, such as the head, fins, and bones. These materials contain many organic compounds such as proteins and lipids, which if utilized as fertilizers could reduce the environmental impacts associated with chemical materials [6,7,8]. In addition, marine wastes have potential as a source of biomass energy when converted by microfloral communities, e.g., methane and hydrogen production by anaerobic fermentation [9,10,11]. Thus, it is important to obtain information regarding the various chemical compositions in marine wastes and the perturbations of metabolic profiles on input-output responses by microfloral communities in soils and anaerobic fermentation processes.

In our previous studies, biomass degradation processes were evaluated under anaerobic conditions to understand anaerobic fermentation by microfloral communities. We revealed the relationship of the degradation of carbon resources (glucose-based materials) with some microbes under anaerobic conditions [12]. Moreover, the metabolic dynamics of anaerobic microfloral communities were also evaluated in terms of metabolic and biogas production and structural changes associated with biomass degradation using ^13^C-labeled bacterial cellulose [13,14]. Although these studies focused on the degradation of sugar-based materials (especially cellulose) by microfloral communities, the metabolic dynamics and anaerobic digestion of protein-rich materials with a low C/N ratio such as fishery products by the microbial communities remains a critical issue for the utilization and recycling of marine wastes.

Considerable tracts of agricultural land are still abandoned in the Tohoku region, Japan, because of a tsunami caused by the 2011 Tohoku earthquake. The inundation caused by the tsunami has strongly affected the soil communities with a high total inorganic nitrogen and ammonium concentration that lasted at least one year [15]. Soil fertilization using an organic fertilizer may provide a useful solution to amend the soil conditions and improve the taste of agricultural products [16,17], because chemical fertilizers are well known to affect the environment negatively when applied for long periods [18]. In this regard, the rice-fish coculture ecosystem is one of the greatest nitrogen recycling systems [19]. Therefore, we attempted to evaluate and amend the abandoned agricultural soils using plant biomass degradation by microfloral communities in Tohoku soil, as demonstrated in previous studies [20,21]. These studies also focused on plant biomass degradation by soil microfloral communities under anaerobic conditions. Amino acid metabolism based on the utilization of marine wastes to improve and fertilize abandoned field soils was not investigated, despite the fact that tons of fish waste is produced in the Tohoku region.

**Figure 1:**
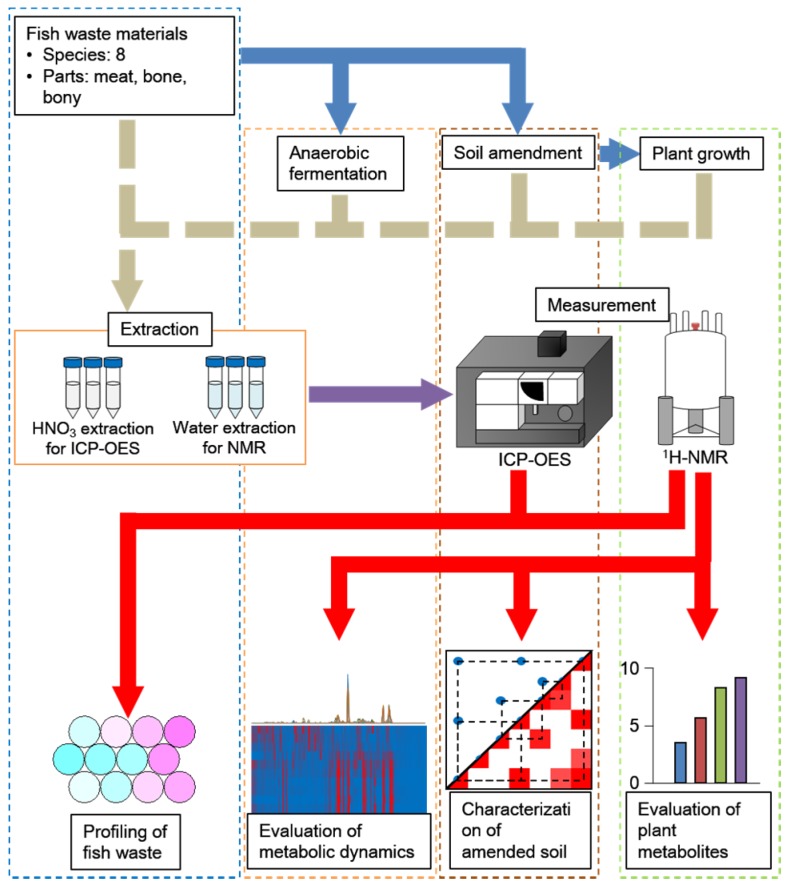
Schematic flow of this study. Sample preparation, those measurements and data visualization are shown from top to bottom, whereas four kinds of evaluation systems are aligned vertically, respectively.

The objective of this study was to characterize soil metabolites by multiple NMR (nuclear magnetic resonance) measurements and to evaluate variations of chemical compositions in fish wastes as input sources for anaerobic microfloral communities. For this purpose, we evaluated the microfloral perturbations by their metabolic responses to chemical variations in the input sources. In this way, we obtained and visualized information about the metabolic dynamics of microfloral communities based on protein-rich substrates with a low C/N ratio in the anaerobic degradation process and in abandoned agricultural soils (Figure 1).

## 2. Results and Discussion

### 2.1. Characterization of Fish Waste

**Figure 2:**
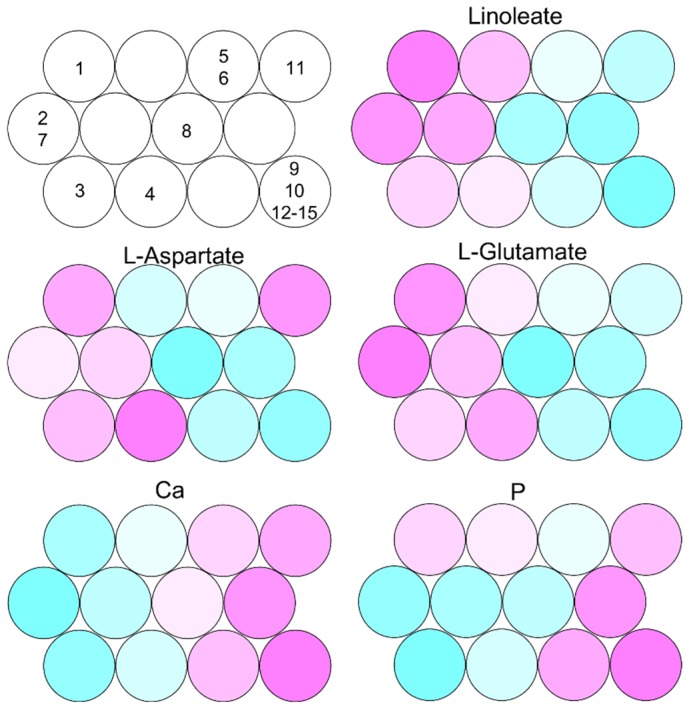
SOM visualization of fish meat and waste material profiles based on integrated data from NMR and ICP-OES: 1. escolar meat; 2. oilfish meat; 3. red stingray meat; 4. left-eyed flounder meat; 5. yellowtail snapper meat; 6. Korean rockfish meat; 7. snake mackerel meat; 8. smooth dogfish meat; 9. escolar fin; 10. oilfish fin; 11. red stingray fin; 12. left-eyed flounder fin; 13. yellowtail snapper fin; 14. Korean rockfish fin; and 15. snake mackerel fin.

Chemical compositions of fish meats and waste materials (fins) were analyzed using ^1^H-NMR and inductively coupled plasma-optical emission spectrometry (ICP-OES) according to previous studies [22,23]. The obtained NMR and ICP-OES data were integrated and further analyzed using the Self-Organizing Map (SOM) approach (Figure 2).

Meats of escolar, oilfish, red stingray, and snake mackerel clustered into the left side of the figure. Meats from oilfish and snake mackerel were clustered together. In contrast, all fin profiles clustered together into the right side in the figure, except for the red stingray fin, which clustered individually to the right. The meat profiles of left-eyed flounder, yellowtail snapper, Korean rockfish, and smooth dogfish lay midway between the other meats and the fins. This suggested that meat and fins accumulated different metabolites and minerals. Indeed, some amino acids such as glutamate were abundantly included in the meats compared to in the fins. Moreover, some lipids such as linoleate were abundantly included in the meats of escolar, oilfish, and snake mackerel compared to the other meats and the fins. Naturally, Ca and P were abundantly included in the fins compared to the meats. These results revealed that the variations and diversities of chemical compositions in the meats were greater than in the fish fins, suggesting that fish wastes may be less variable compared to the meats in metabolic processes of fish wastes by anaerobic microfloral communities, making it easier to deal with the handling of industrial processes using microbial communities.

### 2.2. Metabolic Dynamics of Fish Waste by Microfloral Degradation

**Figure 3:**
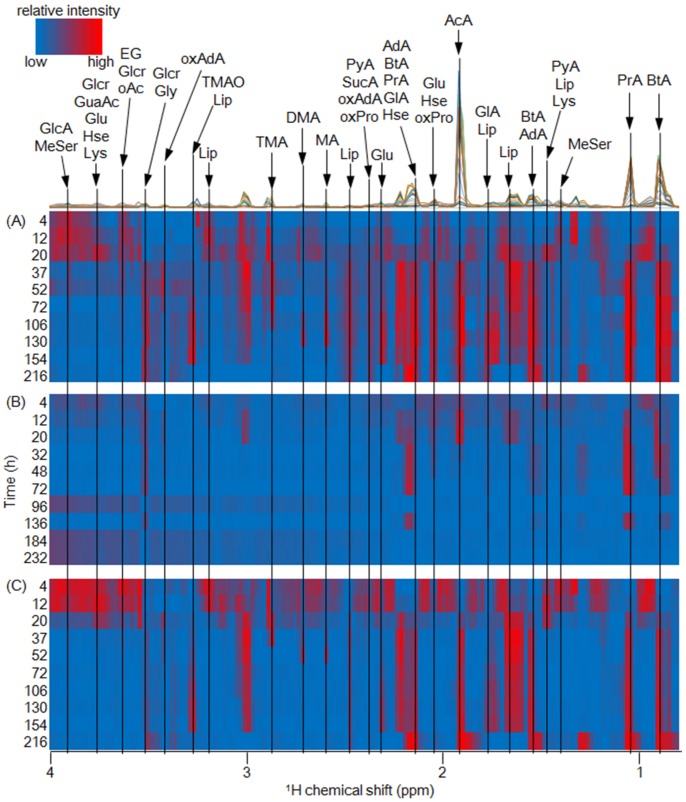
Visualization of metabolic dynamics of fish meat (**A**); fins (**B**); and bony parts (**C**) of left-eyed flounder in anaerobic microfloral digestion processes. Red and blue color denotes high and low intensities of the NMR signals, respectively.

Metabolic dynamics in the microfloral degradation processes of fish meats and waste materials (fins and bony parts) were evaluated using ^1^H-NMR measurements (Figure 3 and Figure S1). For the metabolic annotations of detected peaks in the ^1^H-NMR spectra, statistical total correlation spectroscopy (STOCSY) was performed according to a previous study [24] (Figure S2 and Table S1).

Many weak intensity signals were observed in the meat, fins, and bony parts of left-eyed flounder; for example, some metabolites such as dimethylamine, trimethylamine, and glycine disappeared 20 h after the experiments started. In contrast, stronger intensity peaks assigned as acetate, butyrate, and propionate, and lower magnetized signals which were assigned as phenol compounds, increased from 20 h to the final sampling point. In addition, other fish waste samples digested under anaerobic conditions revealed similar trends between meat and fin samples (Figure S1). Therefore, we surmised that certain metabolites and some amino acids abundant in meat and the bony parts of fish waste were utilized by the microfloral communities, and the microbial communities then produced organic acids such as acetate and propionate under anaerobic conditions. The observed organic acids are important materials that are utilized by microfloral communities as a substrate and play a role in microbial processes, such as hydrogen and methane fermentation. Thus, our results suggested that the fish waste materials (*i.e.*, fins and bony parts) have potential as a source of biomass energy for re-utilizing and recycling. In addition, relatively similar metabolic profiles on output reactions were observed in spite of large chemical variations in input samples (muscles, fins, and bony parts), indicating that microbial communities have a wide tolerance and stable reactions in terms of the metabolic profiles in anaerobic degradation processes of protein-rich materials with a low C/N ratio.

### 2.3. Amendment of Abandoned Agricultural Soils with Plant Growth

**Figure 4:**
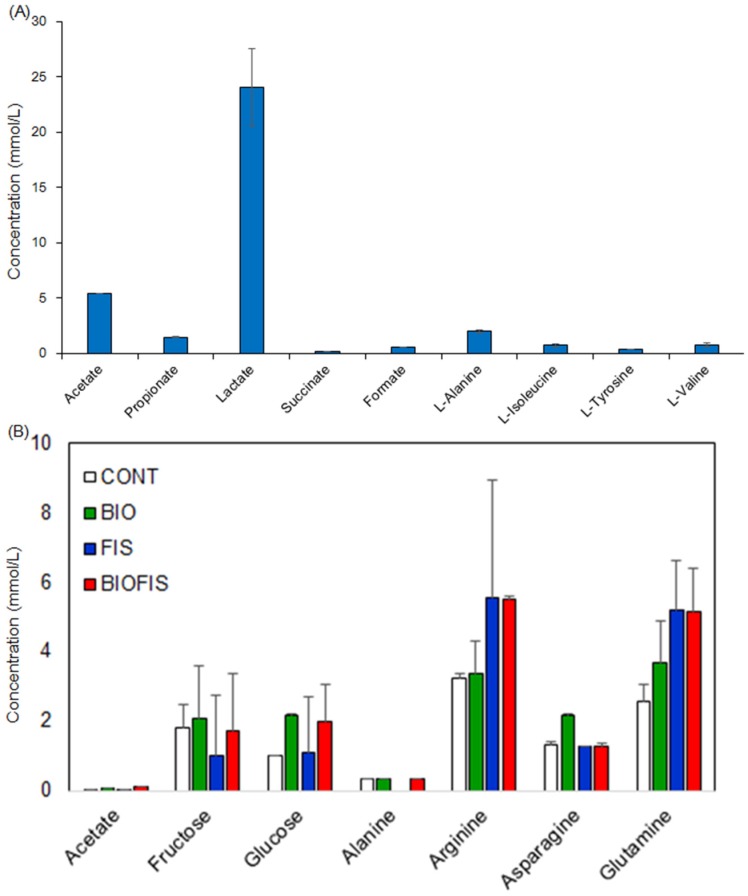
Evaluation of concentration of organic compounds accumulated in soil (**a**) and Komatsuna (**b**). The samples of control, amended soil with biomass, fish, and biomass plus fish were noted as CONT, FIS, BIOFIS, respectively.

Metabolites of soil fertilized with fish waste were measured using ^1^H-NMR and annotated using the HSQC and 2D-*J*res NMR analyses (Table S1 and Figures S3 and S4). Amino acids, including l-isoleucine, l-lysine, and l-threonine, and some organic acids, including acetate, propionate, and butyrate, as well as some other compounds were annotated. To our best knowledge, little information about the annotation of soil metabolites by multiple NMR measurements was reported in previous studies. Therefore, the qualitative annotation result should stimulate further investigation for many researchers. Abundance of metabolites in the soils and accumulation of metabolites in Japanese mustard spinach (Komatsuna), *Brassica rapa*, during the growth experiments were calculated by NMR signal intensities (Figure 4).

Some amino acids such as alanine, valine, and tyrosine were detected in the soil that had added fish waste, and they disappeared within one week after the experiment started. Komatsuna fertilized with fish waste accumulated higher concentrations of l-arginine and l-glutamine compared with the CONT (control) plants. In particular, l-glutamine doubled in concentration and l-arginine was significantly increased compared with the CONT plants. Moreover, there were increasing trends for glucose in BIOFIS (torrefied biomass plus fish added) plants compared with CONT plants. These results suggested that some metabolites and amino acids derived from the input source were directly assimilated by the Komatsuna plant or metabolized and converted by the soil microfloral community followed by the assimilations from the converted metabolites to the Komatsuna. Previous reports regarding plant metabolic profiling also suggested that soil environmental changes induced different metabolite accumulation in plants [25,26]. Therefore, amending abandoned agricultural soils using fish waste is effective for the accumulation of organic compounds in plants. 

We evaluated the effects of fish waste as a marine waste on the anaerobic degradation process and on the soils with Komatsuna. The observed metabolic dynamics were organic acid productions converted from protein and amino acid degradations. Similar degradation and production processes are also observed in cases of animal carcass disposal using livestock animals such as pig andcattle [27], suggesting that the production of organic acids such as acetate and propionate from proteins and amino acids is a common process between marine and terrestrial wastes. Therefore, our visualization method described here was considered to be applicable to the evaluation of metabolic processes in anaerobic microfloral digestions and in soils using both livestock animals and marine wastes. Moreover, although metabolic dynamics were visualized in terms of simple systems such as cell-culture, model animals until now [28,29,30,31,32,33], our visualization method was focused not on model organisms but on real-world data with complex microfloral ecosystems. Therefore, we have established a useful analytical method to visualize metabolic dynamics of microfloral communities for recycling fish waste.

## 3. Experimental Section

### 3.1. Sample Preparation

Fish samples (escolar, oilfish, left-eyed flounder, yellowtail snapper, Korean rockfish, snake mackerel, red stingray, and smooth dogfish) were collected from Suruga Bay, Sagami Bay, Sendai Bay, Off Ohara, and Tokyo Bay in Japan from 2012 to 2013 (Figure S5). All fish samples were dissected into muscle and bony parts including the head, fins, and bones. The samples were freeze-dried for1–2 days, and then milled using a food cutter and a ballmill machine, as described in a previous study [21], for anaerobic microfloral degradation experiments. The freeze-dried bony parts of flounder were milled using a food cutter and then autoclaved for soil amendment experiments. Sawdust of *Cryptomeria* was torrefied at 240 °C for 10 min using an electric furnace for the soil amendment experiments, as essentially similar to previous report [34].

### 3.2. Microfloral Degradation Experiments by Inputting with Fish Waste

Microfloral communities were derived from sediments collected from the Tsurumi river (35°48′86.48″ N, 139°68′00.04″ E), Natori River (38°17′54.25″ N, 140°95′98.58″ E), and anaerobic sludge from a sewage treatment plant (35°48′54.45″ N, 139°41′20.63″ E). The mixture of these sediments and sludge was mixed with artificial sea water and food waste slurry as a substrate for the microbes.The mixture was incubated with each milled fish sample at 40 °C in a 250 mL reactor. Sludge samples (2 mL) were collected from the reactors at 4, 12, 20, 37, 52, 72, 106, 130, 154, and 216 h after the experiment started.

### 3.3. Soil Amendment and Plant Growth Using Abandoned Agricultural Soils in the Tohoku Area

Abandoned agricultural soils were collected from Arahama (38°4′36.72″ N, 140°90′52.46″ E) and riddled using a 5 mm mesh screen. The soils (1.34 kg each) were incubated with MilliQ water and no soil conditioner (control, CONT) or 66.85 g torrefied biomass (BIO) at 60 °C in an incubator for 20 days. Sampling was performed for 40 g every five days during the incubations. After incubations, the soils were divided into four small sized pots and komatsuna was planted into both the CONT and BIO series. For plant growth experiments, 450 mg of the bony parts of left-eyed flounder was added into two pots of the CONT and BIO series (FIS and BIOFIS, respectively). The sampling was performed for 40 g of soils every week.

### 3.4. NMR Measurements

The collected samples from anaerobic microfloral degradation processes and soil amendment experiments were analyzed using ^1^H Watergate and 2D-*J*res NMR spectra for evaluation of metabolic dynamics by microbial communities. NMR spectra were acquired at 25 °C using a 700 MHz (AVANCE II 700) Bruker Biospin (Rheinstetten, Germany) instrument equipped with an inverse (*i.e.*, with proton coils positioned nearest to the sample) 5 mm ^1^H/^13^C/^15^N cryoprobe. The peak of sodium 2,2-dimethyl-2-silapentane-5-sulfonate (DSS) was used as the internal reference (δ_H_ 0 ppm). For anaerobic microfloral degradation experiments, watergate spectra were measured at 25 °C using 32 k data points with 32 scans and eight dummy scans in spectral widths of 9804 Hz. For soil amendment and plant growth experiments, watergate spectra were measured at 25 °C using 32 k data points with 64 scans and two dummy scans in spectral widths of 11,161 Hz. The 2D-*J*res spectra were measured using 16 k (F2) and 16 data points (F1) with 32 scans and 16 dummy scans in spectral widths of9804 (F2) and 50 Hz (F1). The NMR data were processed using Topspin 3.1 (Bruker) and binning about 0.02 ppm width between 11 and −1 ppm with exclusion of water resonance (4.5–5 ppm) using R software [35]. NMR peaks were annotated using the SpinAssign program [36,37].

### 3.5. Elemental Analysis by ICP-OES (Inductively Coupled Plasma-Optical Emission Spectrometry) Measurement

Inorganic elements were analyzed using an ICP-OES (SPS5510; SII Nanotechnology Inc., Tokyo, Japan) instrument according to previous studies [38,39,40].

### 3.6. Statistical Analysis

Data sets obtained from NMR and ICP-OES measurements were integrated and normalized according to previous studies [21,41]. The processed data set was analyzed by SOM on R software according to previous studies [42,43]. Briefly, the SOM was performed using R package “kohonen” with the following parameters: grid, 3 × 4 hexagonal; rlen, 100; alpha, c(0.05, 0.01); init, random; toroidal, false; n.hood, circular.

## 4. Conclusions

In the present study, we evaluated the metabolism of digested marine waste under anaerobic conditions and the fertilization of abandoned agricultural soils using integrated analytical methods. Fish waste profiles were separated into meat and fin parts and their different chemical components affected anaerobic amino acid digestion. Moreover, soil fertilization using fish waste accumulated low levels of compounds derived from fish waste and resulted in amino acid accumulation in plant seedlings. In conclusion, we have established an analytical method to visualize metabolic dynamics of microfloral communities for recycling fish waste.

## Supplementary Materials

The following are available online at www.mdpi.com/2218-1989/6/1/7/s1, Table S1. List of metabolites identified from 1H, 2D-Jres, 1H-13C HSQC spectra, and STOCSY, Figure S1. Visualization of metabolic dynamics of fish meat (A) and fin parts of escolar (B), meat (C) and fin parts of oilfish (D); and meat (E) and fin parts of red stingray (F) in anaerobic microfloral digestion processes, Figure S2. Annotation of produced metabolites from fish waste by anaerobic fermentation using STOCSY, Figure S3. Annotation of accumulated metabolites in amended soil using 2D-Jres NMR spectrum. The region was 0.5–1.5 (A); 1.8–3 (B); 3–4.2 (C) and 6.5–8.5 ppm (D), respectively, Figure S4. Annotation of soil metabolites using 1H-13C HSQC spectrum, Figure S5. Sampling sites of fishes used in this study.
