# Supplementary material for: Visualization of Microfloral Metabolism for Marine Waste Recycling
Source: Metabolites. 2016 Jan 27;6(1):7. doi: 10.3390/metabo6010007 (PMC4812336; doi:10.3390/metabo6010007)
Supplement: Supplementary file 1 [file metabolites-06-00007-s001.pdf]

Supplementary Materials: Visualization of Microfloral Metabolism for Marine Waste Recycling

Tatsuki Ogura, Reona Hoshino, Yasuhiro Date and Jun Kikuchi

Table S1. List of metabolites identified from ^{1}H, 2D-*J*res, ^{1}H-^{13}C HSQC spectra, and STOCSY.

Metabolites	Abbreviations	Hit Rate	^{1}H(multiplicity)/^{13}C Chemical Shift (ppm)	Experiments
2-Methylserine	MeSer	2/3	1.39(m), 3.90(m)	A
2-Oxoglutarate	oxGlA	2/2	2.44(t)/33.44, 3.01(t)	P
3-Oxoadipate	oxAdA	2/3	2.38(m), 3.42(m)	A
5-Oxoproline	oxPro	2/4	2.04(d), 2.38(m)	A
Acetate	AcA	1/1	1.92(s)/26.06	A, S, P
Adipate	AdA	2/2	1.55(m)/28.65, 2.18(m)/40.13	A, S
Betaine	Bet	2/2	3.26(t)/55.98, 3.89(s)/69.37	A, S, P
Butyrate	BtA	3/3	0.87(t), 1.54(m), 2.15(t)	A
Choline	Cho	3/3	3.19(s), 3.53(dd), 4.05(m)	A, S, P
Choline phosphate	ChoP	3/3	3.22(s), 3.59(t), 4.13(m)	A, P
Creatine	Cre	2/2	3.03(s)/39.58, 3.93(s)/56.52	A, S
D-Arabitol	Ara	5/5	3.56(dd), 3.64(m)/65.27,3.75(m)/73.19, 3.84(dd)/65.40, 3.93(m)/63.90	A, S
D-Galactose	Gal	8/9	3.48/74.43, 3.65(m)/75.38, 3.69/77.71, 3.71(m)/63.36, 3.82(m)/71.69, 3.91/71.42, 3.97/71.83, 4.58(d)/99.16	P
D-Glucose	Glc	11/13	3.22(t)/76.89, 3.39(t)/72.24, 3.45(dd)/78.53, 3.52(dd)/73.88, 3.70(dd)/75.38, 3.71(dd)/63.36, 3.81/63.36, 3.81/74.15, 3.88(d)/63.36, 4.63(d)/98.61, 5.22/94.78	P
Dimethylamine	DMA	1/1	2.71(s)/37.26	A, S
Ethanolamine	ETA	2/2	3.12(s), 3.84(s)/60.35	A, P
Ethanolamine phosphate	ETAP	1/2	3.22(t)/43.14, 3.98(dd)	A, P
Ethylene glycol	EG	1/1	3.63(s)/65.14	A, S, P
Formate	FoA	1/1	8.45(s)	S
Gentiobiose	Gen	11/16	3.22/76.89, 3.39(dd)/72.24, 3.45(m)/71.97, 3.45(m)/78.53, 3.52(m)/73.88, 3.70/75.38, 3.71(m)/63.36, 3.82/71.69, 3.88(m)/63.36, 4.63/98.61, 5.22(d)/94.78	P
Glutarate	GlA	2/2	1.78(m), 2.18(t)	A
Glycerol	Glcr	3/3	3.54(m)/65.14, 3.63(m)/65.14, 3.77(m)/74.70	A, S, P
Glycine	Gly	1/1	3.55(s)/44.23	A, S
Glycolate	GlcA	1/1	3.93(s)/63.90	A, S
Guanidinoacetate	GuaAc	1/1	3.78(s)	A
Lactate	LcA	2/2	1.32(d)/22.78, 4.11(q)/71.28	A, S
L-Alanine	Ala	2/2	1.47(d)/18.95, 3.78(q)/53.24	A, S
L-Arginine	Arg	4/4	1.64(m)/26.61, 1.91(m)/30.30, 3.22(t)/43.14, 3.77(t)/56.80	A, P
L-Asparagine	Asn	3/3	2.87(m)/37.26, 2.94(m)/37.26, 3.99(dd)/53.80	P
L-Glutamate	Glu	3/4	2.11(m)/29.88, 2.35(q)/36.17, 3.75(d)/57.34	A, S
L-Glutamine	Gln	3/3	2.13(m)/28.79, 2.44(m)/33.44, 3.77(dd)/56.80	A, P
L-Homoserine	Hse	3/4	2.04(d), 2.16(m), 3.78(m), 3.84(dd)	A
Linoleate	LA	6/7	0.88(t), 1.27(m), 1.32(d), 1.54(mn), 2.04(d), 2.72(t)	A
Lipoamide	Lip	6/8	1.62(m), 1.66(m), 1.74(m), 3.2(m), 3.26(t), 3.72(m)	A
L-Isoleucine	Ile	4/6	0.93(t), 1.00(d), 1.47(m), 3.65(d)	A, S
L-Leucine	Leu	4/5	0.95(t)/23.73, 0.96(d)/24.69, 1.72/42.45, 3.73/56.11	S
L-Lysine	Lys	5/5	1.48(m), 1.72(m), 1.89(m), 3.01(t), 3.75(t)	A
L-Serine	Ser	2/2	3.84(t)/58.85, 3.95(m)/62.81	A, P
L-Threonine	Thr	3/3	1.32(d), 3.59(d)/62.95, 4.23/68.55	A, P
L-Tyrosine	Tyr	5/5	3.02(m), 3.2(m), 3.92(m), 6.86(d), 7.18(d)	A, S
L-Valine	Val	4/4	0.99(d)/19.36, 1.03(d)/20.73, 2.28/31.93, 3.60(d)	A, S
Methylamine	MA	1/1	2.60(s)	A
Methyl ethyl ketone	MEK	2/3	0.99(s), 2.18(s)	S
N1-Acetylspermine	AcSpd	6/6	1.78(s), 1.91(q), 2.00(s), 2.12(m), 3.03(m), 3.26(t)	S
Oxaloacetate	OAc	1/1	3.64(s)	A
Propionate	PrA	2/2	1.05(t)/12.94, 2.18(q)/33.43	A, S
Putrescine	Put	2/2	1.75(m), 3.02(t)	A
Pyruvate	PyA	1/1	2.32(s)	A

Table S1. *Cont.*

Metabolites	Abbreviations	Hit Rate	[1]H(multiplicity)/[13]C Chemical Shift (ppm)	Experiments
Raffinose	Raf	6/12	3.52(t), 3.71(t)/63.36, 3.82(m)/71.69, 3.88(m)/72.24, 3.97(t)/71.83, 5.41(d)	P
Ribitol	Rib	3/4	3.64(dd)/65.27, 3.78(dd)/65.27, 3.78(dd)/74.83	S
Suberate	SubA	2/3	1.32(s), 2.18(t)	S
Succinate	SucA	1/1	2.40(s)/36.85	
Sucrose	Suc	5/11	3.45(dd)/71.97, 3.52(dd)/73.88, 3.77(m)/74.70, 3.89(m), 5.41(d)	P
Taurine	Tau	2/2	3.26(t)/50.24, 3.41(t)/38.08	A, S
Trimethylamine	TMA	1/1	2.9(s)	A
Trimethylamine N-oxide	TMAO	1/1	3.26(s)/62.26	A, S
Uridine	Uri	7/8	3.81(dd)/63.36, 3.88(dd)/63.36, 4.11(q)/86.86, 4.21(dd)/72.10, 4.33(m)/76.20, 5.86(m)/105.03, 5.89(m)/91.91	P

Multiplicity: s = singlet, d = doublet, dd = doublet of doublets, t = triplet, m = multiplet; Experiments: A = anaerobic fermentation, S = soil amendment, P = plant growth.

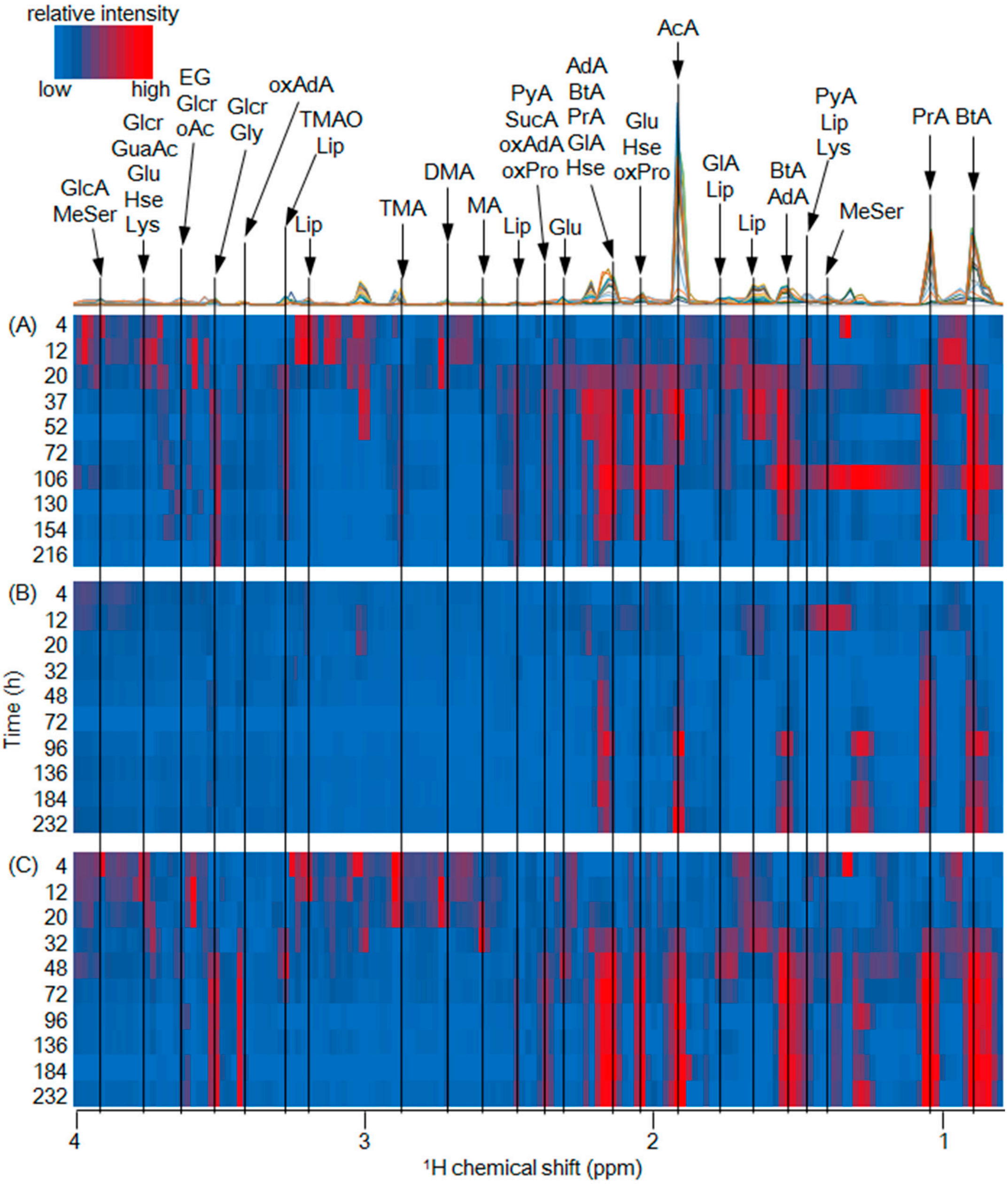

Figure S1. *Cont.*

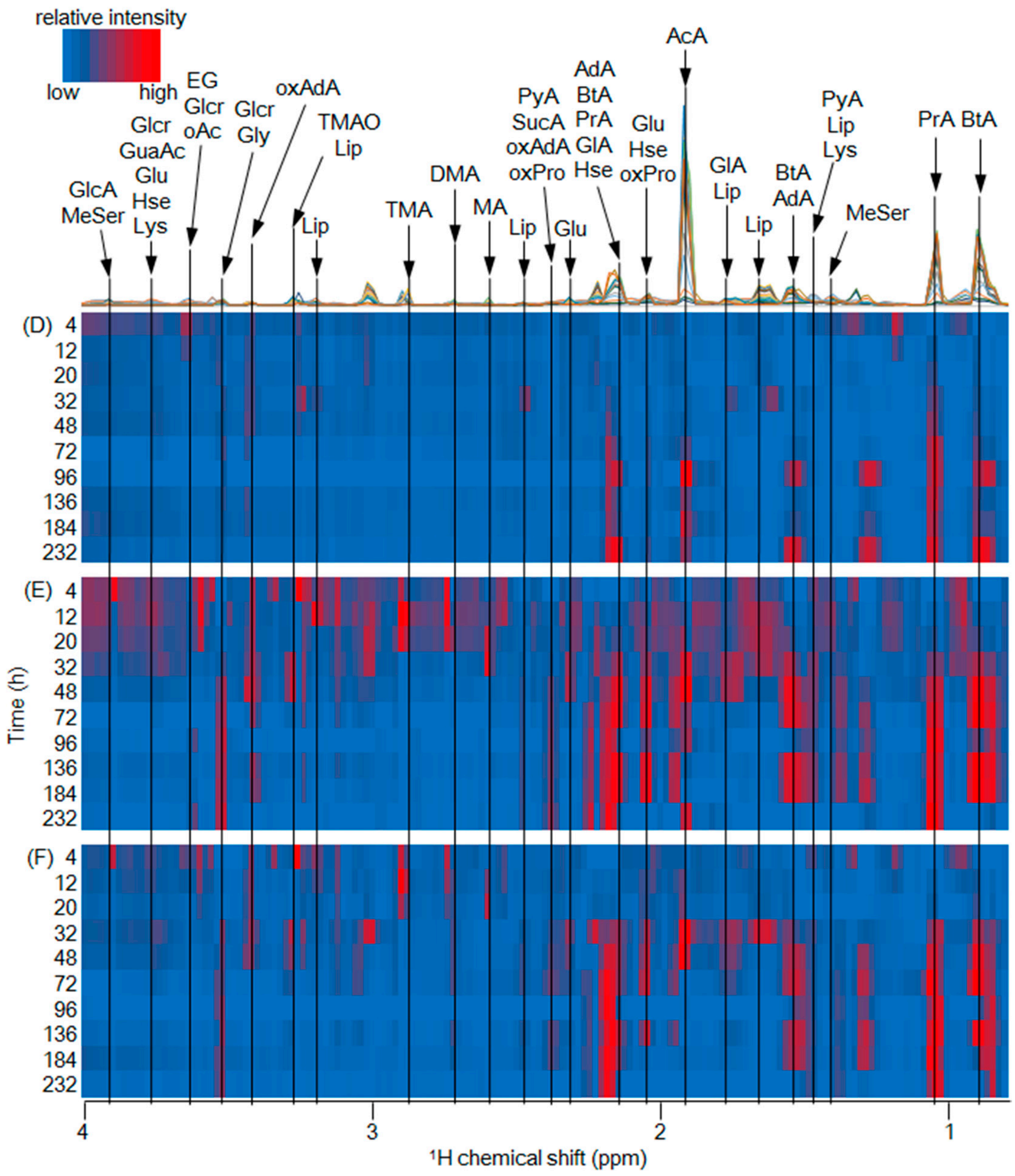

Figure S1. Visualization of metabolic dynamics of fish meat (**A**) and fin parts of escolar (**B**); meat (**C**) and fin parts of oilfish (**D**); and meat (**E**) and fin parts of red stingray (**F**) in anaerobic microfloral digestion processes. Red and blue color denotes high and low intensities of the NMR signals, respectively.

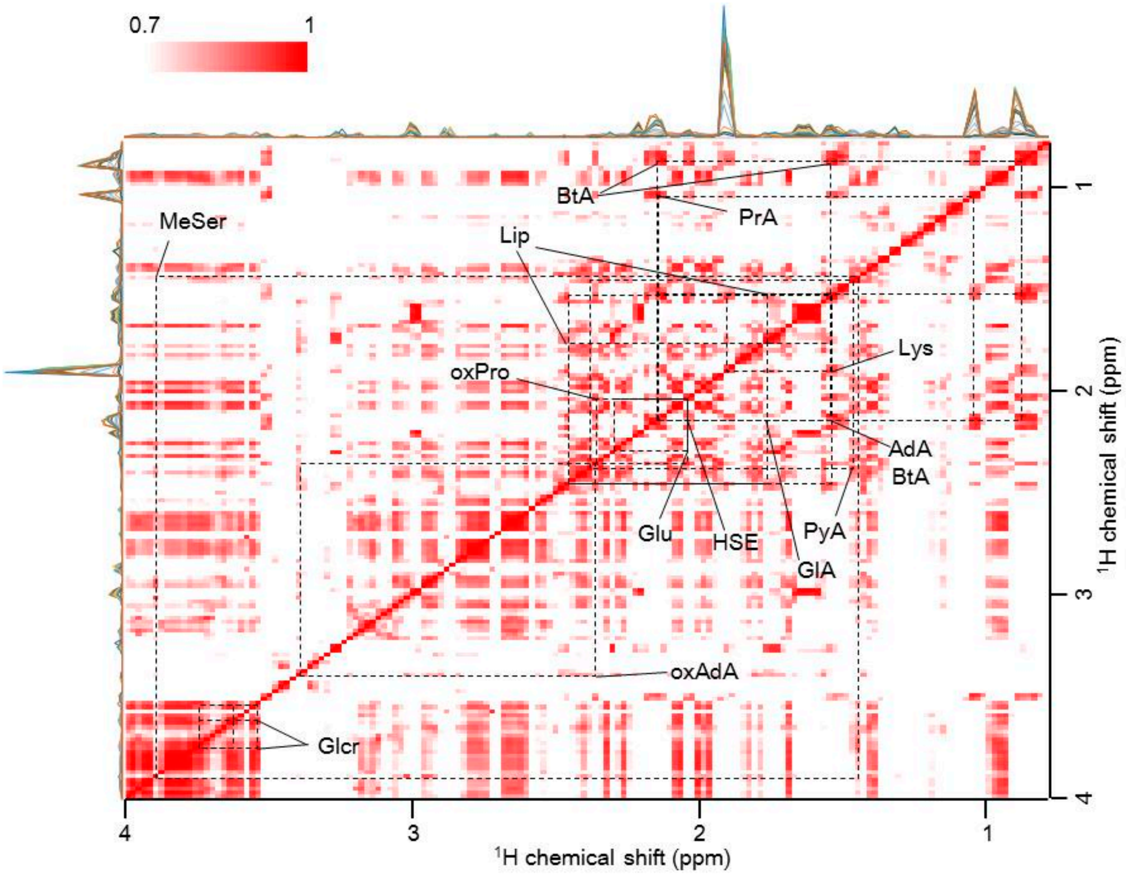

Figure S2. Annotation of produced metabolites from fish waste by anaerobic fermentation using STOCSY. Red color code highlighted top indicate correlation coefficient from 0.7 to 1.0.

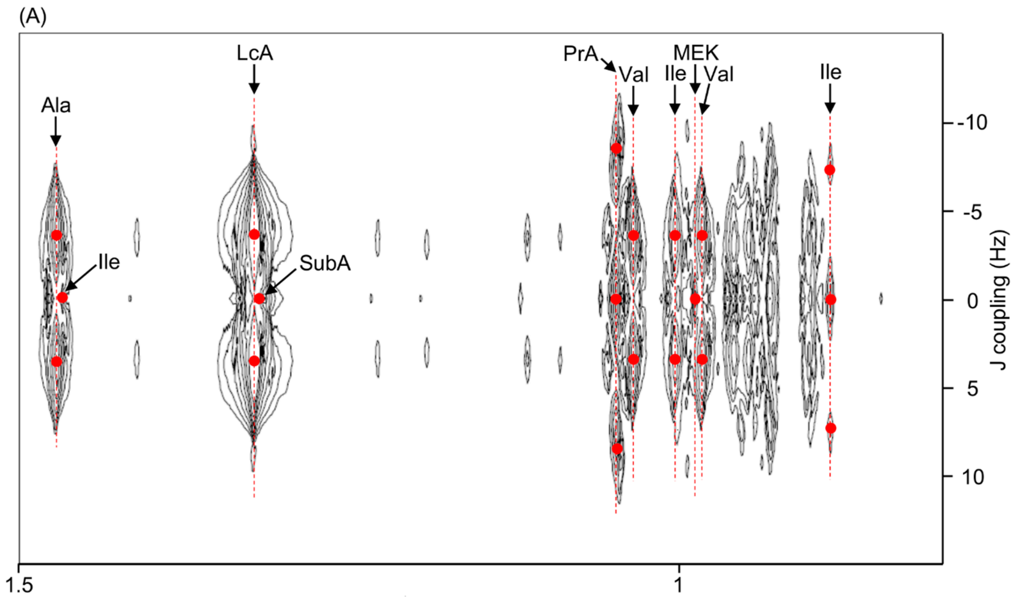

Figure S3. *Cont.*

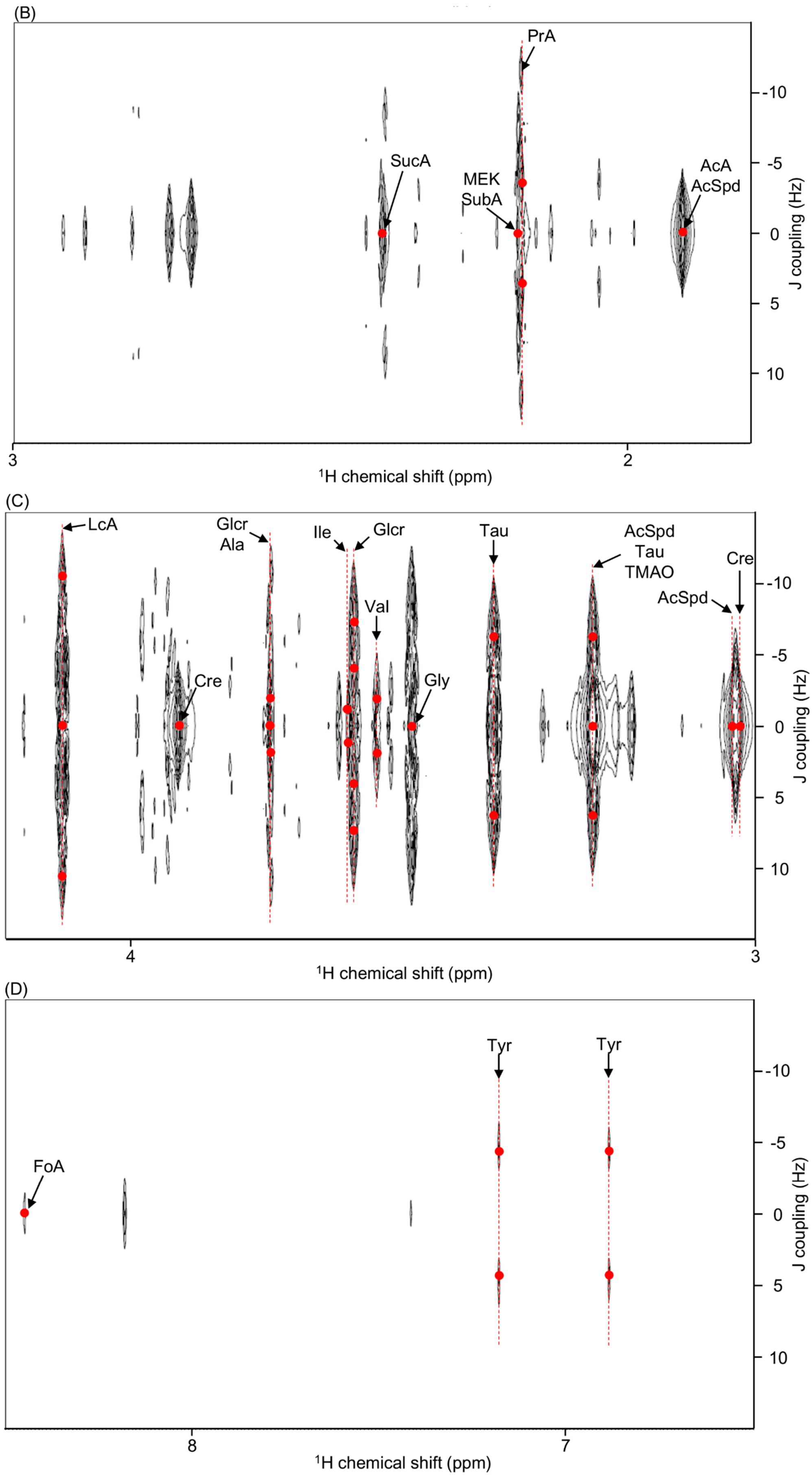

Figure S3. Annotation of accumulated metabolites in amended soil using 2D-*J*res NMR spectrum. The region was 0.5–1.5 (**A**); 1.8–3 (**B**); 3–4.2 (**C**) and 6.5–8.5 ppm (**D**), respectively.

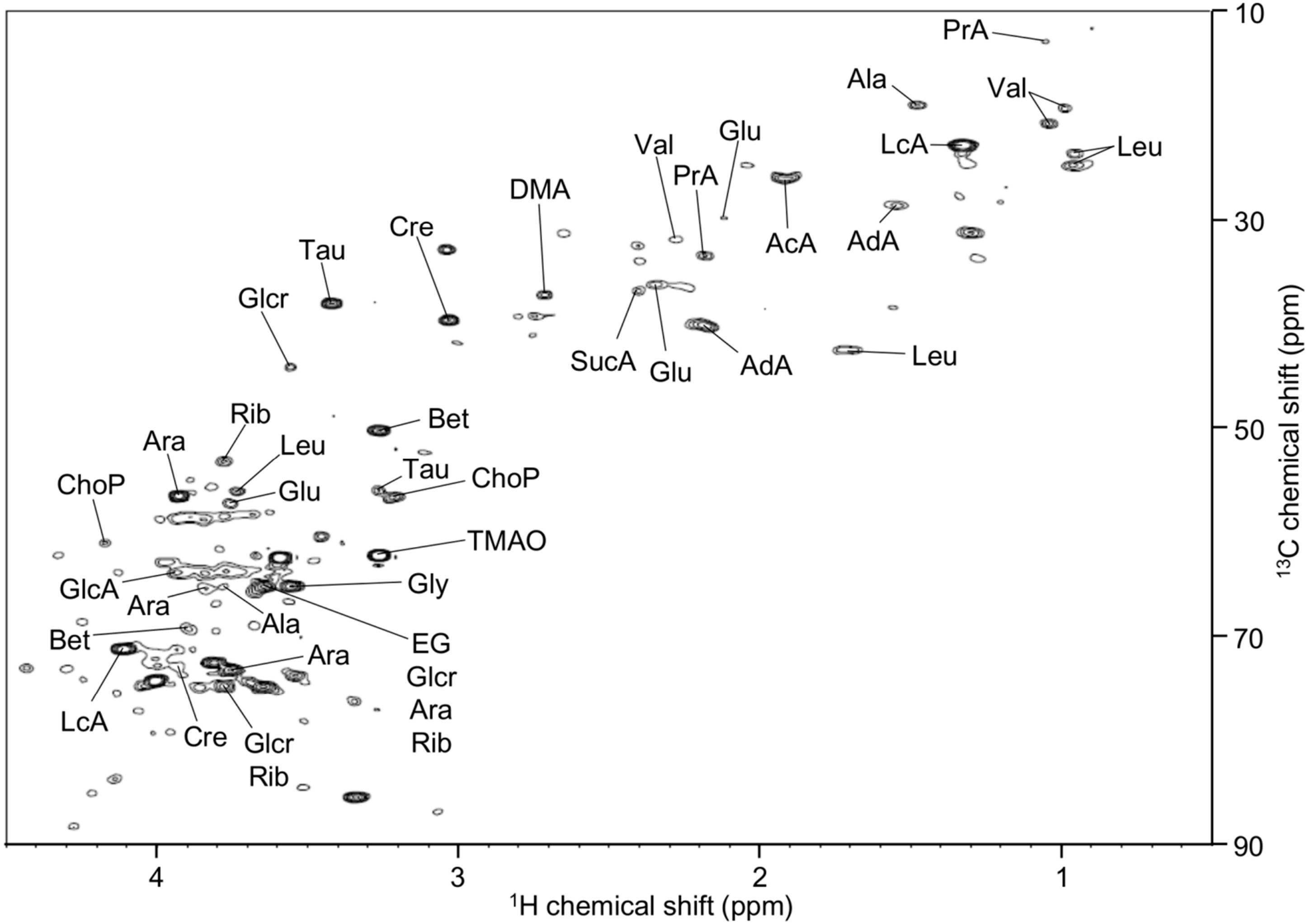

Figure S4. Annotation of soil metabolites using ^{1}H-^{13}C HSQC spectrum.

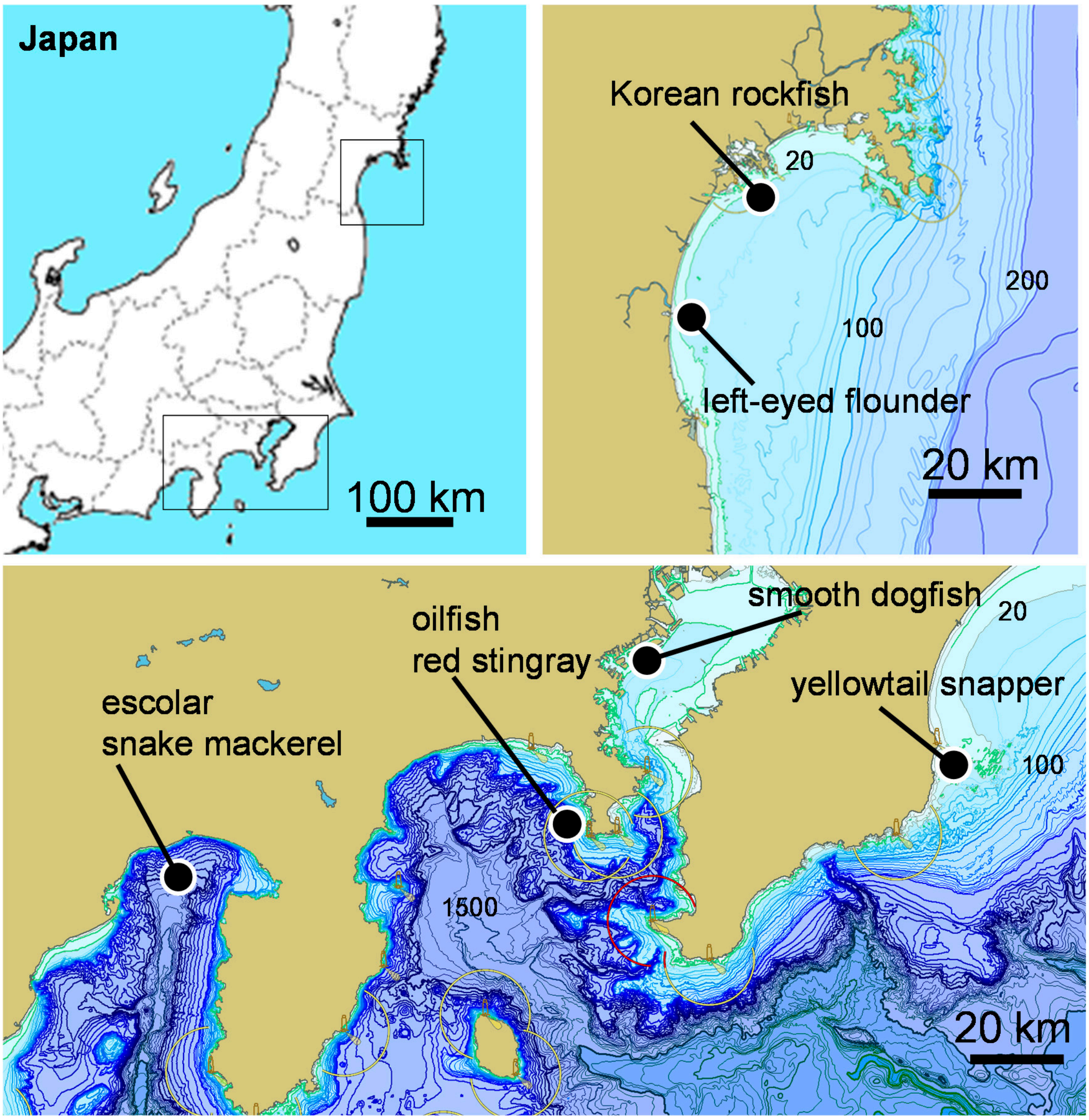

Figure S5. Sampling sites of fishes used in this study. Numbers highlighted in coastal region are the depth of the water.
